# Supplementary material for: Chromothripsis during telomere crisis is independent of NHEJ, and consistent with a replicative origin
Source: Genome Res. 2019 May;29(5):737–49. doi: 10.1101/gr.240705.118 (PMC6499312; doi:10.1101/gr.240705.118)
Supplement: Supplemental Material [file supp_gr.240705.118_Supplemental_file_1.zip › contigs/annotated_contigs/DB53/contig.3.DB53_length_282_mean_cov_3.72340425532.docx]

**DB53_length_282_mean_cov_3.72340425532**

```
ACAGAATAACCTATGGCAGAGACTGCCGGTGCATGACCAA|A|GGCAGGCAACAGGCATTGGAAATGTTTATTGTAAGGTCTAATACAA
 >chr8:84469531-84469572 - E=2e-13       >chr6:47024919-47025107 - E=4e-102
TTCTCAAATTTTTTTGCATTTGTACTGTTTACTATACAAAATTGTCAGCTCTCTATATCCTGGGACTTGGTATACTTCTTACTGTGTCT

GACACAGTACTTACCTTAGTTCTGGGATCATCTACTAAAACAGGCACTGAGG|AAATAAACTTTCCATGGCAATCTATTTTCTGAAGAC
                                                      >chr17:68747096-68747150 + E=4e-21
TTTTGCTTTGTTTGAATT
```
